# Socioeconomic inequality in stunting among under-5 children in Ethiopia: a decomposition analysis

**DOI:** 10.1186/s13104-019-4229-9

**Published:** 2019-03-29

**Authors:** Shimels Hussien Mohammed, Fatima Muhammad, Reza Pakzad, Shahab Alizadeh

**Affiliations:** 1 Department of Community Nutrition, School of Nutritional Sciences and Dietetics, Tehran University of Medical Sciences-International Campus, Tehran, Iran; 2 Department of Epidemiology and Biostatistics, School of Public Health, Tehran University of Medical Sciences-International Campus, Tehran, Iran; 3 Department of Epidemiology, Faculty of Health, Ilam University of Medical Sciences, Ilam, Iran; 4 Noor Research Center for Ophthalmologic Epidemiology, Noor Eye Hospital, Tehran, Iran; 5 Department of Clinical Nutrition, School of Nutritional Sciences and Dietetics, Tehran University of Medical Sciences, Tehran, Iran

**Keywords:** Socioeconomic inequality, Stunting, Ethiopia

## Abstract

**Objective:**

Ethiopia bears a high stunting burden. However, there is a paucity of evidence on the socioeconomic inequalities of stunting in Ethiopia. Thus, this study was aimed to determine the degree of socioeconomic inequality in stunting and decompose it to the social determinants of stunting. We used a nationally representative sample of 8855 children, aged below 5 years, from the Ethiopian demographic and health survey, conducted in 2016. Subjects were recruited following a two-stage cluster sampling. The socioeconomic status was measured by the household wealth index, categorized into quantiles. The inequality in stunting between the poorest and the richest socioeconomic groups was decomposed into its contributing social factors following the Blinder–Oaxaca decomposition approach.

**Result:**

The overall prevalence of stunting was 38%, with a significant pro-poor socioeconomic inequality. The prevalence of stunting among the poorest and the richest socioeconomic categories was 45.1% and 26.9%, respectively. Caregivers education status was the main contributor, accounting alone for 33% of the socioeconomic inequality in stunting, followed by region of residence (11%) and birth size (6%). Equity sensitive interventions, which prioritize the vulnerable groups might help to narrow the socioeconomic inequality as well as fasten the progress towards the goal of stunting reduction.

## Introduction

Stunting, defined by linear growth failure and reflecting chronic malnutrition, remains a major threat to the health and survival of children globally [1]. Stunted children are at higher risk of developing physical, cognitive, and mental health impairments during childhood as well as during later stages of life [1, 2]. Thus, the prevention of stunting has been adopted as one of the six main global nutrition targets for the period until 2025 [3]. Several studies in low and middle-income countries have shown that socioeconomic inequality is linked to stunting, such that communities with high socioeconomic inequality bear a disproportionately high burden of stunting [4, 5]. Thus, the issue of socioeconomic equality has taken a centrality in the launching of the sustainable development goals, which calls upon nations to narrow socioeconomic inequalities in health [6].

Decomposition approaches help to break down the health/nutrition inequality into its driving factors, thereby estimating the independent contribution of each of the determinants to the observed socioeconomic inequality [7]. Understanding the contribution of the drivers of socioeconomic inequality in health and nutritional outcomes could guide the design of equity sensitive health and nutrition interventions [7, 8].

Ethiopia bears one of the highest burdens of stunting. Despite the last two decades has seen a decline in the proportion of stunted children in Ethiopia, the rate of decline has been slow compared with the international standard [9]. There is also a paucity evidence on socioeconomic inequalities of stunting in Ethiopia. The degree of the inequality and the contribution of the driving factors to the stunting inequality, if any, have not yet been documented. Thus, this study was aimed to determine the extent of socioeconomic inequality in stunting among under-5 Ethiopian children and to decompose the inequality into its social determinant factors. We used a nationally representative sample from the Ethiopian demographic survey, conducted in 2016 (EDHS 2016).

## Main text

### Methods

#### Population and data source

For this work, we used the data of children included in EDHS 2016. All children included in the survey were below 5 years of age. DHS surveys are one of the most cited databases globally and have been conducted in over 90 countries since 1984 [10]. In Ethiopia, the DHS program has been conducted every 5 years since 2000, managed financially by the United States Agency for International Development (USAID) and technically by ICF International, in collaboration with the Ministry of Health of Ethiopia [9].

#### Sample size and sampling strategy

EDHS 2016 was a national survey conducted following a multistage sampling strategy. Census enumeration areas served as primary sampling units during the first stage of sample selection. In total, 645 clusters were selected randomly. Households served as secondary sampling units. From each selected cluster, a fixed number of 28 households were selected randomly. In total, we found 8855 children under 5 years of age included in the dataset and with complete information on the variables of interest [9].

#### Inclusion and exclusion criteria

The main inclusion criteria for this study were age 0 to 59 months, and data on height (length), and wealth index measured and indexed with the child profile. Exclusion criteria were incomplete data on age, length (height), wealth category, and implausible height-for-age (HFA) Z-score values. Implausible HFA Z score values was defined by HFA Z-score < − 6 or > 6, according to the WHO 2006 child growth standards [9].

### Variables and measurements

#### Stunting

The length of children under-2 years of age was measured in a recumbent position and the height of those 2 years and above was measured in standing position. Using the length, height, and age data, length-for-age (LFA Z-score) was calculated for children under-2 years of age and height-for-age (HFA) Z-score for those 2 years and above. The WHO 2006 child growth standard was used as a reference in the calculation of both LFA and HFA Z-scores [9]. Except for the age group, both LFA and HFA Z-score refer to the linear growth status of children as compared with the median of the reference population [11]. As HFA is more familiar and commonly used in nutritional reports, HFA is used in the subsequent sections of this report to refer to both LFA and HFA Z-scores. Stunting, or linear growth failure, is defined when HFA is below − 2 Z-scores [11].

#### Socioeconomic status

The socioeconomic status of children was measured by the household wealth index, developed by principal component analysis. The wealth index was categorized into quantiles (poorest, poorer, middle, richer, and richest).

#### Determinants of stunting

Though stunting is of multiple risk factors originating from various levels, this work was limited to its social determinants.

- Place of residence: urban or rural.
- Region: agrarian or pastoral.
- Caregivers education status: illiterate, primary, or secondary and above.
- Water sources: improved or unimproved.
- Toilet facility: improved or unimproved.
- Child sex: boy or girl.
- Child age: < 24 months or 24–59 months.
- Birth size: small, average, or medium.

### Statistical analysis

All analyses took were done taking into account the complex design of the survey. Thus, all estimates reported were based on the weighted sample (n = 9588), not on the unweighted sample (n = 8855). The weighting was done to adjust for the inequality in sampling probability due to the over-representation of small states (regions) [9]. Adjustment for design effect was also done to account for the variance inflation due to the cluster sampling strategy. All analyses were done using STATA 15 software, with statistical significance determined at P ≤ 0.05. The prevalence of stunting by the five socioeconomic groups as well as by the determinant factors was estimated by the proportion of those with HFA < − 2 Z-scores. The relationship between the determinant variables and stunting was determined by doing bivariate analyses, using the Chi square test of association. The socioeconomic gap in stunting between the richest and the poorest groups was decomposed into its contributing social determinants, following the Blinder–Oaxaca decomposition approach, specifically the Blinder–Oaxaca decomposition for nonlinear regression models [12].

### Result

A total of 8855 (9588 weighted) under-5 children with complete data on the variables of interest were found in the dataset and included in this analysis. Boys were 51.9% and girls 48.1%. The age of the children ranged from 0 to 59 months, with mean (SD) of 27.89 (11.06) months. The children aged 2 years and above constituted for 58.2% and those below 2 years for 41.8% of the sample. Overall, 38.4% of the children were stunted. The distribution of stunting by five socioeconomic categories as well as by the social determinant variables is shown in Table 1. The results of the bi-variable analyses between the determinant factors and stunting are also shown in the same table. There was a significant variation in stunting by socioeconomic groups (P < 0.001). The prevalence of stunting among the poorest and the richest wealth categories was 45.1% and 26.9%, respectively. There was also a significant difference in the prevalence of stunting by place of residence, region of residence, caregivers’ education status, water source, toilet facility, sex, age, and birth size (P < 0.001).

**Table 1:** Relation of basic and underlying factors with stunting (weighted n = 9588)

Variables	Weighted frequency (%)	Stunting prevalence (95% CI)	P*
*Wealth category*			
Poorest	23.9	45.1 (41.5–48.6)	< 0.001
Poorer	22.9	44.1 (40.5–47.6)	
Middle	20.7	40.7 (37.2–44.3)	
Richer	18.1	33.3 (30.0–36.5)	
Richest	14.4	26.9 (23.8–30.0)	
*Residence place*			
Urban	10.9	26.7 (23.3–30.2)	< 0.001
Rural	89.1	39.9 (38.2–41.6)	
*Region (state)*			
Agrarian	93.0	38.4 (36.8–40.0)	< 0.001
Pastoral	7.0	31.3 (26.4–36.2)	
*Household size*			
< 4	10.5	39.0 (34.2–43.8)	0.353
4–8	76.4	37.6 (35.9–39.3)	
> 8	13.1	37.8 (33.4–42.2)	
*Caregivers education status*			
Illiterate	66.1	42.0 (40.0–43.9)	< 0.001
Primary	26.8	33.4 (30.5–36.3)	
Secondary+	7.1	20.9 (16.7–25.1)	
*Water source*			
Not improved	50.9	39.8 (37.7–41.9)	< 0.001
Improved	49.1	35.3 (33.1–37.6)	
*Toilet facility*			
Not improved	90.9	39.1 (37.4–40.7)	< 0.001
Improved	9.1	27.9 (23.7–32.3)	
*Child sex*			
Boy	51.9	41.8 (39.6–44.0)	< 0.001
Girl	48.1	33.7 (31.6–35.8)	
*Child age (months)*			
< 24	41.8	29.7 (27.6–31.7)	< 0.001
24–59	58.2	45.1 (43.0–47.1)	
*Birth size*			
Small	26.2	44.3 (41.1–47.5)	< 0.001
Average	41.9	38.5 (36.2–40.8)	
Large	31.9	31.9 (29.3–34.5)	

*CI* confidence interval

* P: based on Chi square test of association

The result of the Blinder–Oaxaca decomposition analysis of stunting inequality between the richest and the poorest wealth categories is shown in Table 2. The difference between the richest and the poorest categories was 18.22%. Of the overall inequality, 61.12% was due to compositional factors, i.e. due to the difference in the social determinants of stunting between the richest and the poorest categories. The unexplained inequality, i.e. due to coefficients, was 38.88%. The compositional factors found contributing to the stunting inequality significantly were region (state) of residence, caregivers’ education status, and birth size (P < 0.05). Among these, caregivers’ education status made the largest contribution to the socioeconomic inequality in stunting, accounting for 33.33% of the inequality alone (Coefficient = 0.06, P = 0.003). State (region) of residence accounted for 11.11% of the inequality (Coefficient = − 0.02, P = 0.010). Small birth size contributed to 5.56% of the socioeconomic inequality in stunting, indicating a higher proportion of low birth weight among the poor than the rich (Coefficient = 0.01, P = 0.047).

**Table 2:** Decomposition of difference in stunting between the richest and the poorest socioeconomic groups

Variable	Difference due to characteristics (E)			Difference due to coefficients (C)		
	Coefficient	Percent	P	Coefficient	Percent	P
Residence place	0.04	22.22	0.167	− 0.01	− 5.56	0.001
Region (state)	− 0.02	− 11.11	0.010	− 0.06	− 33.33	0.413
Caregivers education	0.06	33.33	0.003	− 0.03	− 16.67	0.418
Water source	− 0.00	0.00	− 0.38	− 0.01	− 5.56	0.834
Toilet facility	0.01	5.56	0.296	− 0.00	0.00	0.982
Child sex	− 0.00	0.00	0.915	0.08	44.44	0.401
Child age	0.01	5.56	0.230	0.06	33.33	0.150
Birth size	0.01	5.56	0.047	− 0.03	16.67	0.736
Constant				0.07	38.89	0.002
Total	0.11	61.12	< 0.001	0.07	38.88	0.013

### Discussion

We found a significant socioeconomic inequality in stunting among under-5 children in Ethiopia. Caregiver’s education status, region of residence, and birth size were the significant contributors to the socioeconomic inequality in stunting in Ethiopia.

Our finding of an overall 38.4% prevalence of stunting was consistent with the findings of previous studies which consistently reported a two-fifth national prevalence of stunting in Ethiopia in 2016 [9, 13]. There was a significant discrepancy in the proportion of stunted children across the five socioeconomic categories. The inequality was pro-poor socioeconomic, such that the lower socioeconomic groups were more likely to be stunted and bear a higher burden of the problem than the higher socioeconomic groups. Our findings were consistent with the reports of previous studies done in similar settings. Studies that were done in Kenya [14], Bangladesh [14, 15], and Iran [16] also reported a pro-poor socioeconomic inequality in stunting among under-5 children. Generally, stunting is a pro-poor condition particularly in developing countries [17]. There are many plausible explanations for the link of socioeconomic inequalities to stunting, including a low nutritional literacy, poor dietary and hygiene practices among the poor compared to the rich [18, 19]. Our finding that maternal education status accounted for the largest proportion of the richest-poorest difference in stunting was also consistent with the findings of previous studies. For example, Emamian et al. reported as maternal education was the single most important factor in socioeconomic inequality in stunting among children aged 6 to 59 months [16].

The study has both research and policy implications. Despite many studies have shown that socioeconomic inequality is associated with stunting, it is, however, unclear if narrowing socioeconomic inequality reduces the burden of stunting. Socioeconomic inequality may be low while the average health and nutritional services are low. Thus, narrowing socioeconomic inequality does not necessarily equate with a quality child health care [7]. Specific to stunting, further studies are needed to know which approach, narrowing inequality or improving general health service, is a more feasible and relevant approach. Meanwhile, the findings of this study imply the importance of mapping and targeting the socioeconomic groups vulnerable to stunting in Ethiopia.

In conclusion, we found a significant pro-poor socioeconomic inequality in stunting among under-5 children in Ethiopia, with an 18% stunting gap between the richest and the poorest socioeconomic groups. Caregivers’ education status, region (state) of residence, and birth size were the main contributing factors to the gap in stunting between the richest and the poorest socioeconomic groups.

## Limitations

The cross-sectional design of the study precludes making causal inference on whether high socioeconomic inequality in itself leads to stunting. It is also worth noting to the reader that narrowing socioeconomic inequality might not necessarily reduce the burden of stunting as the absolute mean socioeconomic status may be more important to promote optimal child nutrition and health status. All the determinants of stunting were not included in this analysis, which might have limited the comprehensiveness of the findings.

## Abbreviations

CI: confidence interval
EDHS: Ethiopian demographic health survey
HFA: height-for-age
LFA: length-for-age
WHO: World Health Organization
